# Prevalence of Autoimmune Pancreatitis and Other Benign Disorders in Pancreatoduodenectomy for Presumed Malignancy of the Pancreatic Head

**DOI:** 10.1007/s10620-012-2191-7

**Published:** 2012-05-16

**Authors:** M. J. van Heerde, K. Biermann, P. E. Zondervan, G. Kazemier, C. H. J. van Eijck, C. Pek, E. J. Kuipers, H. R. van Buuren

**Affiliations:** 1 Department of Gastroenterology and Hepatology, Erasmus University Medical Center, PO box 2040, 3000 CA Rotterdam, The Netherlands; 2 Department of Pathology, Erasmus University Medical Center, Rotterdam, The Netherlands; 3 Department of Surgery, Erasmus University Medical Center, Rotterdam, The Netherlands; 4 Department of Internal Medicine, Erasmus University Medical Center, Rotterdam, The Netherlands

**Keywords:** Autoimmune pancreatitis, Autoimmune cholangitis, IgG4, Pancreatoduodenectomy, IgG4 related systemic disease

## Abstract

**Background:**

Occasionally patients undergoing resection for presumed malignancy of the pancreatic head are diagnosed postoperatively with benign disease. Autoimmune pancreatitis (AIP) is a rare disease that mimics pancreatic cancer. We aimed to determine the prevalence of benign disease and AIP in patients who underwent pancreatoduodenectomy (PD) over a 9-year period, and to explore if and how surgery could have been avoided.

**Methods:**

All patients undergoing PD between 2000 and 2009 in a tertiary referral centre were analyzed retrospectively. In cancer-negative cases, postoperative diagnosis was reassessed. Preoperative index of suspicion of malignancy was scored as non-specific, suggestive, or high. In AIP patients, diagnostic criteria systems were checked.

**Results:**

A total of 274 PDs were performed for presumed malignancy. The prevalence of benign disease was 8.4 %, overall prevalence of AIP was 2.6 %. Based on preoperative index of suspicion of malignancy, surgery could have been avoided in 3 non-AIP patients. All AIP patients had sufficient index to justify surgery. If diagnostic criteria would have been checked; however, surgery could have been avoided in one to five AIP patients.

**Conclusions:**

The prevalence of benign disease in patients who underwent PD for presumed malignancy was 8.4 %, nearly one-third attributable to AIP. Although misdiagnosis of AIP as carcinoma is a problem of limited quantitative importance, every effort to establish the correct diagnosis should be undertaken considering the major therapeutic consequences. IgG4 measurement and systematic use of diagnostic criteria systems are recommended for every candidate patient for PD when there is no histological proof of malignancy.

**Electronic supplementary material:**

The online version of this article (doi:10.1007/s10620-012-2191-7) contains supplementary material, which is available to authorized users.

## Introduction

Nowadays, routine work-up consists of CT scan, frequently combined with endoscopic ultrasonography (EUS) and fine needle aspiration (FNA) cytology. Although the sensitivity of EUS with FNA is approximately 80 % and the specificity of positive cytology approaches 100 % [1], false negative results are common and the negative predictive value of these tests is low [2]. Therefore, if a person presents with a mass in the pancreatic head without metastases, a PD will usually be considered, as it is the only curative option. Five to 11 % of patients however are found to have a benign disease on postoperative histological examination [3–8]. In large volume centers the mortality of this operation is less than 5 % [9] and morbidity is a substantial 46 % [4].

Autoimmune pancreatitis (AIP) is a rare disease that may present with a pancreatic head mass, jaundice, and weight loss, and thus may mimic pancreatic carcinoma clinically. Biliary involvement (distal and proximal) is common, sometimes without overt pancreatic disease, mimicking cholangiocarcinoma. The disease is highly responsive to steroids [10], and this feature can be used as a diagnostic tool [11]. The exact pathogenesis is unknown. In 68–95 % of patients, IgG4 serum levels are elevated [12–16]. AIP can be associated with extrapancreatobiliary manifestations like retroperitoneal fibrosis, Sjögren’s disease, rheumatoid arthritis, inflammatory bowel disease, interstitial nephritis, thyroïditis, or inflammatory tumors in lungs, mediastinum, or liver. According to several large retrospective series, 23–38 % of benign PDs are due to autoimmune pancreatitis [3, 4]. Increasing knowledge and awareness of this intriguing disease is expected to avoid unnecessary surgery in a substantial number of patients. Unfortunately, there is no single diagnostic test. Several diagnostic criteria systems of AIP have been proposed, including the HISORt and Asian criteria [17, 18]. The aims of this study were first to determine the prevalence of benign disease and in particular of AIP in patients who underwent PD for presumed malignancy in the past decade, second to investigate if there was any decline in misdiagnosis over time, and third to assess if and how unnecessary surgery possibly could have been avoided.

## Methods

### Study Population

All patients undergoing PD between January 1, 2000 and January 31, 2009 in a tertiary referral center with multidisciplinary approach to pancreatic and biliary disease were retrospectively analyzed. Patients were included if the indication for surgery was suspicion of malignancy in the pancreatic head. If postoperative diagnosis did not harbor a benign or malignant neoplasm, it was classified as a benign PD. Demographic characteristics (age, gender, and mortality) were evaluated in all patients. In benign PDs, postoperative diagnosis was reassessed by revision of histological and clinical data.

The following clinical data were extracted from patient case records: age, gender, diabetes mellitus, history of chronic pancreatitis, autoimmune disease, smoking, alcohol consumption, jaundice, weight loss, and pain. Laboratory results of bilirubin, Ca19-9, total IgG, IgG4, and autoantibodies (RF, ANF) were recorded. Relevant radiological and endoscopic studies (ultrasound US, computed tomography CT, magnetic resonance imaging MRI, endoscopic retrograde cholangio pancreatography ERCP, endoscopic ultrasound EUS) were reviewed. Based on these data and—if available—preoperative cytological or histological examination, a preoperative index of suspicion of malignancy (non-specific, suggestive, highly suspicious) [6] was calculated (detailed information in Addendum Table 3). Van Gulik et al. [6] described this system in 1999, using US and ERCP features of malignant and inflammatory lesions in the pancreatic head. We added clinical symptoms (weight loss, jaundice and pain), level of Ca19-9 [19], EUS features [8], and pathology findings (preoperative histology or cytology). For each examination, suspicion of cancer was scored on a 0/+/++ scale. Retrospectively, surgery was considered unnecessary when preoperative findings were non-specific. In AIP patients, the HISORt and Asian diagnostic criteria systems (Addendum Table 4) were applied on preoperative data, to determine if and how surgery could have been avoided.

### Histopathologic Evaluation

Resection specimens were revised by two expert pathologists familiar with pancreatic disease and with special interest in AIP. Immunostaining for IgG4 was performed using a monoclonal mouse anti-human IgG4 (Zymed Laboratories, San Francisco, USA), with a working dilution of 1:100. The presence of >10 IgG4-positive plasma cells in at least one HPF at a magnification of ×400 was considered suggestive of AIP. Each specimen was evaluated for the presence of microscopic AIP features, as previously established in several series of resection specimens [20–27]. A classical histological triade is recognized in 80 %: dense lymphoplasmacytic infiltration, cuff-like periductal fibrosis, and obliterative phlebitis (venulitis). Other common features are: perineural inflammation, acinar atrophy or fibrosis, storiform (spindle shaped) fibrosis, granulomas, and the presence of neutrophils and eosinophils. More recently, two subtypes of autoimmune pancreatitis have been distinguished, each with a distinct clinical and histopathological picture: the predominant lobular type (AIP-PL or type 1) and the predominant ductal type (AIP-PD, type 2) [21]. AIP type 1 represents the “classic” lymphoplasmacytic sclerosing pancreatitis, more prevalent in older men, and is strongly associated with retroperitoneal fibrosis and biliary strictures, the latter often becoming prominent after pancreaticoduodenectomy. Especially, this type of AIP is associated with an elevated serum IgG4 and the presence of IgG4 positive plasma cells in tissue. The less well-known AIP type 2 is characterized by the presence of so-called GELs: granulocytic epithelial lesions, which represent destruction of pancreatic interlobular ductal epithelium [26]. This subtype is more prevalent in younger patients, more often associated with ulcerative colitis or Crohn’s disease and generally shows no recurrence after resection. It is less associated with increase of IgG4. While AIP type 1 usually presents with typical histological pattern, AIP type 2 could be more difficult to diagnose, both preoperatively on biopsy material as well as on resection specimens. The typical fibrosis is missing and IgG4 staining is less useful [20–27].

Suggestive of other forms of chronic pancreatitis are pseudocysts and calcifications, irregular ductal dilation, mucoprotein plugs, and necrosis (suggestive of chronic alcoholic or obstructive pancreatitis), pancreas divisum, or inflammation of the duodenal wall (groove pancreatitis) [3].

### Statistical analysis

Chi square and unpaired *t* test were used to compare gender and age between malignant and benign postoperative diagnosis. Fischer’s exact test and unpaired *t* test were used to compare differences in characteristics and symptoms of patients with benign pancreatoduodenectomies. Two-tailed *p* values of <0.05 were considered statistically significant.

## Results

**Fig. 1:**
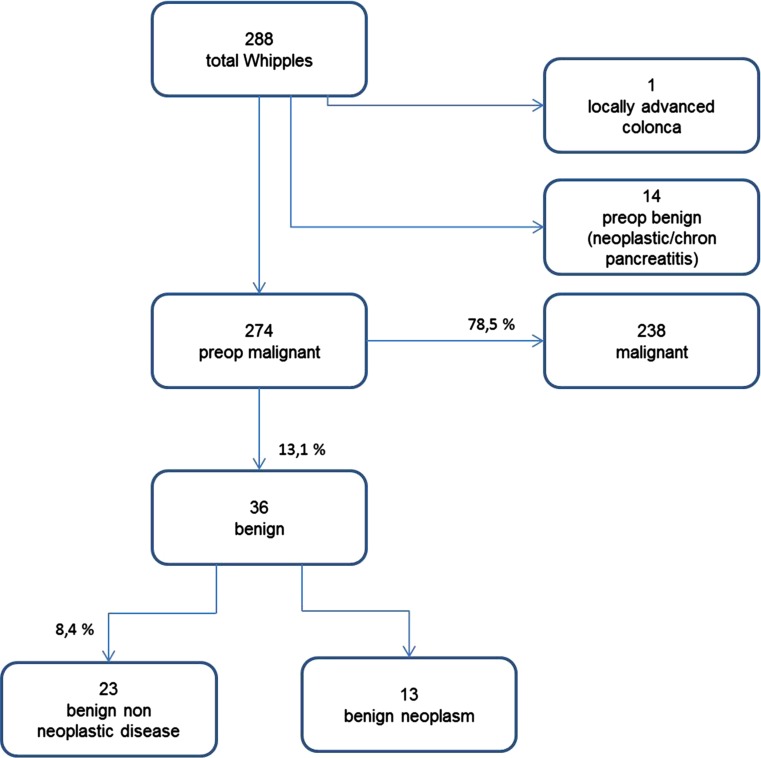
Flow chart of patient inclusion

Of 288 pancreatoduodenectomies performed during 2000–2009, 274 were performed for presumed malignancy. Twenty-three (8.4 %) of 274 resections were negative for neoplastic disease (Fig. 1). Patients with malignancy were significantly older (mean 63.7 ± 10.1 years) than those with benign disease (mean 58.6 ± 12.7) (*p* = 0.004). There was no difference in gender (*p* = 0.832). Overall, operative mortality was 20/288 (6.9 %) but mortality was not observed in the benign PD cases. Mortality did not differ between the first and second half of the study period (7.1 vs. 6.8 %, *p* = 1.0).

In Table 1, postoperative diagnoses of 23 benign PDs are summarized. AIP was diagnosed in 30.4 %, that is 2.6 % of total PDs performed for presumed malignancy. Clinical characteristics and symptoms of benign PDs (AIP and non-AIP) are summarized in Table 2. No statistical differences were noted between AIP and non-AIP except for pre-operative presence of diabetes mellitus, being more frequent in AIP patients (71 vs. 19 %, *p* = 0.026).

**Table 1:** Clinicopathologic classification of disease in 23 benign pancreatoduodenectomies

	No of patients (%)
Chronic pancreatitis	
Alcoholic	3 (13.0 %)
Obstructive	7 (30.4 %)
Idiopathic	3 (13.0 %)
Autoimmune	6 (26.1 %)
Biliary tract disease	
Autoimmune	1 (4.3 %)
Idiopathic	1 (4.3 %)
Papillary fibrosis	1 (4.3 %)
Crohn’s disease (infiltrate)	1 (4.3 %)

**Table 2:** Characteristics and symptoms of patients with benign pancreatoduodenectomy

	AIP	Non-AIP	*p* value
No.	7	16	
M:F ratio	6.0	2.2	0.62
Mean age years (±SD)	53 (±19.7)	54 (±7.9)	0.65
Diabetes (de novo)	5 (2) (71 %)	3 (2) (19 %)	0.03
History of chronic pancreatitis	0	2 (13 %)	1.00
Autoimmune disease	2 (29 %)	1 (6 %)	0.21
Smoking	5 (71 %)	9 (56 %)	0.66
Alcohol > 2 U daily	1 (14 %)	8 (50 %)	0.18
Jaundice	6 (86 %)	7 (44 %)	1.00
Mean weight loss kg (±SD)	2.7 (±5.6)	7.0 (±7.7)	0.21
Pain			
None/mild	5 (71 %)	10 (63 %)	1.00
Moderate/severe	2 (29 %)	6 (37 %)	1.00

Fisher’s exact and unpaired *t* test

*SD* standard deviation

The prevalence of misdiagnosis in the first and second half of the study period showed a decline from 10.9 to 5.8 %, but it failed to gain statistical significance (*p* = 0.19). The proportion AIP among misdiagnosed patients remained constant (26.7 vs. 37.5 %, *p* = 0.66).

Based on the preoperative index of suspicion of malignancy (Table 3), postulating that for surgery findings should at least be suggestive, resection could have been avoided in three non-AIP patients, one with alcoholic and two with obstructive chronic pancreatitis. The index of suspicion in these cases was non-specific. Radiology was indicative of chronic pancreatitis without clear signs of malignancy. The decision to operate was mainly based on symptoms (suggestive *n* = 2 or non-specific *n* = 1). The index of suspicion was also non-specific in another case finally diagnosed with Crohn’s disease, but surgery could possibly not have been avoided since a tumor-like mass was found infiltrating both pancreas and colon ascendens and causing obstructive symptoms. The index of suspicion in all seven patients with AIP was sufficient to justify the operation (suggestive *n* = 4; strong suspicion *n* = 3). Important reasons to operate were marked elevation of Ca19-9 (levels as high as 23 284 kU/l), suggestive imaging (mass on EUS, double duct sign on CT/MRI or ERCP, regional adenopathy on CT or MRI), and (false) positive cytology (EUS-FNA). Based on diagnostic criteria systems for AIP, however (Table 4), surgery could have been avoided in at least one case. This patient developed biliary strictures postoperatively, triggering clinicians to consider AIP. The preoperative IgG4 level (measured retrospectively) was very high (13.6 g/l). Based on the spectacular response to steroids postoperatively, it is very likely that steroids would have prevented the operation. In four patients, findings at pancreatography and/or elevated IgG4 levels would have justified a steroid trial. However, none of the patients had an adequate pancreatogram, and in only one case had IgG4 been measured preoperatively. In two AIP patients, surgery also seemed inevitable in retrospect. Even if responsive to steroids, criteria would not be met (no other criterion present, IgG4 normal). In summary, surgery could have been avoided in at least 4 (which would reduce the percentage benign PDs to 6.9), but possibly 8, patients (three non-AIP and five AIP) according to the index of suspicion for malignancy and the HISORt criteria. The pre-operative work-up in AIP patients was unsatisfactory.

**Table 3:** Preoperative index of suspicion of malignancy and final diagnosis in benign pancreatoduodenectomy

No	M/F	Age (years)	Symptoms	Ca19.9 kU/L	Pathology	Radiology	EUS	Index suspicion	Final diagnosis
1	M	54	Non-specific	<34	n.a.	Neoplasm	n.a.	Suggestive	CAP
11	M	52	Strong	<34	n.a.	Neoplasm	n.a.	Strong	CAP
12	M	59	Suggestive	<34	n.a.	CP	n.a.	Non-specific	CAP
2	F	48	Non-specific	<34	n.a.	Neoplasm and CP	n.a.	Suggestive	CIP
4	M	41	Strong	<34	n.a.	Non-specific	n.a.	Suggestive	CIP
14	M	58	Non-specific	<34	n.a.	Neoplasm	CP	Suggestive	CIP
3	F	50	Suggestive	<34	Benign	CP	n.a.	Non-specific	COP
6	M	55	Strong	n.a.	n.a.	CP	n.a.	Suggestive	COP
8	F	48	Suggestive	<34	n.a.	Neoplasm and CP	n.a.	Suggestive	COP
17	M	57	Strong	50	n.a.	Neoplasm	n.a.	Strong	COP
18	M	71	Strong	<34	n.a.	Neoplasm and CP	n.a.	Strong	COP
20	M	68	Suggestive	1308	n.a.	Neoplasm and CP	CP	Strong	COP
21	F	52	Non-specific	<34	n.a.	Non-specific	n.a.	Non-specific	COP
7	M	75	Suggestive	68	n.a.	Neoplasm	n.a.	Suggestive	AIP type 1
16	M	69	Strong	23284	n.a.	Neoplasm	n.a.	Strong	AIP type 1
15	M	33	Suggestive	<34	n.a.	Non-specific	Neoplasm	Suggestive	AIP type 2
5	M	73	Suggestive	<34	Benign	Neoplasm	n.a.	Suggestive	AIP type 2
10	M	53	Suggestive	1689	Benign	CP	n.a.	Strong	AIP type 2
23	F	28	Suggestive	<34	Malignant	Neoplasm and CP	Neoplasm and CP	Strong	AIP type 2
19	M	40	Suggestive	<34	Benign	Neoplasm	Neoplasm	Suggestive	AIC
9	M	52	Non-specific	n.a.	Benign	n.a.	n.a.	Non-specific	Crohn’s
13	M	66	Strong	<34	Atypical	CP	Non-specific	Strong	IC
22	F	59	Suggestive	<34	Benign	Neoplasm	Non-specific	Suggestive	Papillary fibrosis

*n.a* not available. Ca 19.9 normal <34 kU/L, *CP* chronic pancreatitis, *CAP* chronic alcoholic pancreatitis, *CIP* chronic idiopathic pancreatitis, *COP* chronic obstructive pancreatitis (stones, neoplasm, divisum), *AIP* autoimmune pancreatitis, *AIC* autoimmune cholangitis, *IC* idiopathic cholangitis

**Table 4:** Preoperative findings and work-up in patients diagnosed with AIP after pancreatoduodenectomy: did they meet the diagnostic criteria?

No	Histology postop	Histology preop	Typical imaging (atypical)	IgG	IgG4	AAB	Other organ involvement	Steroid trial	Asian positive^e^	HISORt positive
5	AIP type 2	n.a.	No	n.a.	n.a.	n.a.	No	No	No	Possible^c^
7	AIP type 1	n.a.	No (focal mass)	n.a.	n.a.	n.a.	Hypothyroidism	No	No	Possible^c^
10	AIP type 2	n.a.	No	n.a.	n.a.	n.a.	No	No	No	Possible^c^
15	AIP type 2	n.a.	No (focal mass)	11.8^a^	1.39^a^	n.a.	No	No	No	No^d^
16	AIP type 1	n.a.	No (focal mass)	33.0^a^	13.6^a^	Negative	Retroperitoneal Fibrosis	No	No	Yes
19	AIC	n.a.	Biliary stricture	n.a.	n.a.	n.a.	No	No	No	Possible^c^
23	AIP type2	Malignant^b^	No (diffuse enlargement no rim, focal mass)	8.6	0.05	n.a.	No	No	No	No^d^

IgG normal <17.0 g/L, IgG4 nl <1.40 g/L. *n.a*. not available, *AAB* auto antibodies (RF, ANA)

^a^Preoperative values, measured retrospectively, ^b ^cytology (EUS FNA), ^c ^if serology positive and/or suggestive pancreatogram, responsiveness to steroids would have confirmed diagnosis, ^d ^even if responsive to steroids, diagnostic criteria would not have been met, ^e ^none of the patients had adequate (mandatory) pancreatogram; patients 16 and 19 had double duct sign on ERCP with minimal contrast injection

## Discussion

The prevalence of benign disease in patients who underwent PD for presumed malignancy in our center was 8.4 %. During a 9-year period, seven patients were postoperatively diagnosed with AIP, corresponding with a total prevalence in this population of 2.6 % and accounting for nearly one-third of all benign cases. These findings show that AIP accounts for a significant proportion of incorrect preoperative diagnoses, but also indicate that, from a quantitative perspective, missing the diagnosis of AIP was a problem of limited magnitude. In our national AIP database, containing 130 patients, 20 % underwent resection for presumed malignancy (unpublished data). Our data are compatible with other large series, reporting 5–11 % [3–8] benign disease in patients after PD for suspected malignancy, with AIP constituting 23–38 % of benign cases [3, 4]. The prevalence declined over time, although this was not statistically significant.

A preoperative diagnosis of AIP was missed for several reasons. First, we noted insufficient preoperative work up in patients finally diagnosed with AIP. IgG4 measurements were missing in 6/7 cases and adequate imaging of the pancreatic duct was not performed in any patient, both being crucial elements in either the American (HISORt) or Asian diagnostic strategy [28]. Second, the importance of Ca19-9 was overestimated. Levels of >300 U/ml are thought to be pathognomonic for malignancy [19], but markedly elevated levels were found in two of our AIP patients. The third reason is the mere fact that in some patients it may be virtually impossible to detect the disease without resecting the pancreas. In a recent study, in which a diagnostic strategy to distinguish AIP from pancreatic cancer based on HISORt criteria was tested, researchers from the US found that sensitivity of diagnostic criteria is 70 %*.* In 30 % of AIP cases, however, the diagnosis could not be confirmed without a steroid trial, pancreatic core biopsy, or surgical resection [29].

Based on the index of suspicion of malignancy we used in this study, three non-AIP patients underwent PD while the index was non-specific. Nowadays, we believe that the index should at least be suggestive before embarking on surgery. Although seemingly easy to use, this index is subject to personal interpretation, and discussion about findings to be interpreted as “suggestive” or “very suspicious” is inevitable. To better define the clinical usefulness of the index prospective validation studies are needed. Noteworthy, applying this index illustrated the fact that in patients with AIP findings may clearly suggest malignancy. Unnecessary surgery can be avoided only if this diagnosis is always considered and actively pursued.

Diagnosing AIP may be troublesome. The two main diagnostic systems (HISORt and Asian diagnostic criteria) are based on specific combinations of radiological (focal enlargement, sausage-shaped pancreas with hypodense rim, diffuse or segmental narrowing of the pancreatic duct), serological (IgG4, IgG and the presence of autoantibodies like RF or ANA), and histological (pancreatic and or extrapancreatic tissue) findings, and the response to steroid therapy. An extensive discussion of the diagnostic criteria is beyond the scope of this article, but in preoperative work-up, the following clues are of key importance and should be looked for in every patient: elevated IgG4, narrowing of the pancreatic duct (in contrast with ordinary carcinoma patient who usually presents with double duct sign), and evidence of extrapancreatobiliary involvement.

In our opinion, a diagnostic strategy of measuring serum IgG4 levels in all patients suspected of pancreatic or cholangiocarcinoma could well be considered. Of all patients referred for presumed malignancy, 20 % are candidates for surgery. With a prevalence of 2.5 % among those undergoing PD, 200 patients would need to be screened to detect one case of AIP eligible for surgery. At approximately $50 per test, $10,000 would be spent for each patient preoperatively diagnosed with AIP, an amount considerably less than the costs of surgery and its associated morbidity (about $30,000) [2, 30]. In resection for presumed hilar cholangiocarcinoma, the percentage autoimmune cholangitis is probably higher (1.1–8.1 %) [31] and fewer patients would need to be screened. In our center, with an annual volume of approximately 30 PDs, and taking into account that sensitivity of IgG4 is 68–95 % [12–16], it would take at least one and a half years of routine screening to detect one patient with AIP. Although this may seem a low yield of this screening strategy, this approach may still be defendable and worthwhile in the light of possible unnecessary major surgery, morbidity, and mortality. This strategy would also allow the detection of patients with AIP considered to have irresectable malignancies because of infiltration, lymphadenopathy, or supposed metastases. This group is easily forgotten but not less important or tragic: be diagnosed with incurable cancer while steroids can heal. Although routine IgG4 measurement preoperatively has been gradually introduced in our center since 2006, we have not been able to prevent the one case that was diagnosed postoperatively after 2006. This young female, with preoperative normal IgG4 and a cytology report of malignancy on EUS-FNA, was diagnosed with AIP type 2. It is only recently that AIP type 2 is acknowledged as a distinct phenotype. It is more difficult to detect because IgG4 is often not elevated and patient characteristics are very different from the classical jaundiced old man with weight loss and retroperitoneal fibrosis. This case reflects the lacuna in current diagnostic strategies, especially in IgG4 negative disease. Another important aspect and limitation of measuring IgG4 is that levels up to 2 times the upper limit of normal can also be found in patients with pancreatic cancer, primary sclerosing cholangitis, and other pancreatic disease. The specificity of, in particular, slightly elevated levels is limited [12–16, 32]. If a cut-off value is used of >2.8 g/L, however, specificity rises to 98 % [13, 29].

The second tool to detect AIP preoperatively is histology. In contrast to Asian criteria, the HISORt already diagnoses AIP if only histology is positive. This gives pancreatic core biopsy a special significance. Obviously, reliable histological assessment requires a dedicated pathologist, who is familiar with the histological features of pancreatic disease and IgG4 immunostaining. AIP can usually be diagnosed in resection specimens without great difficulty and be distinguished clearly from other types of pancreatitis and adenocarcinoma. IgG4 immunostaining however has limited sensitivity and specificity and shows overlaps between AIP, chronic pancreatitis and adenocarcinoma. Deshpande et al. showed that IgG4 positive cells were identified in resection specimens in 42.9 % cases of chronic pancreatitis and 52.6 % cases of adenocarcinoma (using a working dilution of 1:50, scored in a 20× field). These findings suggest limited diagnostic value of pancreatic biopsy [21]. Data regarding the role of pancreatic biopsy, however, are sparse and disputed. Detlefsen et al. [33] recognized AIP in pancreatic core biopsies using six microscopic features [granulocytic epithelial lesions (GELs), >10 IgG4-positive per high power field (HPF), >10 eosinophilic granulocytes/HPF, cellular fibrosis with inflammation, lymphoplasmacytic infiltration, and venulitis]. They were able to detect AIP in 76 % when they used a cut-off level of four features, rising to 86 % when cases were added with three features including GELs. In this study, there was no control group with adenocarcinoma. The Mayo Clinic group was able to detect AIP in EUS-guided true cut biopsies in 100 % [34]. Further studies are required to further establish the diagnostic significance of pancreatic biopsy in patients possibly suffering from AIP.

The third major diagnostic tool is pancreatography. Preoperative ultrasound and/or or CT showing a non-dilated pancreatic duct should always give rise to suspected AIP and not cancer. When MRI is performed, MRCP should be performed as well. Although a recently published randomized controlled trial showed that, in carcinoma of the pancreatic head, early surgery is superior to preoperative biliary drainage, most patients will still undergo an ERCP before surgery [35]. While gastroenterologists will usually not try to deliberately cannulate and fill the pancreatic duct, adequate pancreatography is helpful in establishing the correct diagnosis.

Finally, a 2-week trial of corticosteroids [11] can confirm the diagnosis, but this should only be considered if other findings clearly suggest the possibility of AIP. We believe it is an important tool but should be left in experienced hands and only after careful multidisciplinary review of all relevant data. Malignant tumors as well as benign non-autoimmune-mediated inflammatory processes may respond to steroids to some degree, and victims of the autoimmune hype have already been reported [36].

## Conclusions

Prevalence of benign disease in patients who underwent pancreatoduodenectomy for presumed malignancy is 8.4 %. One-third of these cases are diagnosed with AIP. In 9 years, the prevalence of benign PDs showed a non-significant trend towards decline from 10.9 to 5.8 %. The proportion AIP remained stable, at least partially due to insufficient preoperative work-up. Routine work-up for pancreatic cancer is not enough to detect these patients beforehand. IgG4 measurement and systematic use of diagnostic criteria systems should be considered in every patient eligible for PD but without preoperative histological confirmation of malignancy.

## Electronic supplementary material

Below is the link to the electronic supplementary material.

Supplementary material 1 (DOC 39 kb)

Supplementary material 2 (DOC 33 kb)
