# Supplementary material for: Prevalence of Autoimmune Pancreatitis and Other Benign Disorders in Pancreatoduodenectomy for Presumed Malignancy of the Pancreatic Head
Source: Dig Dis Sci. 2012 May 16;57(9):2458–65. doi: 10.1007/s10620-012-2191-7 (PMC3428528; doi:10.1007/s10620-012-2191-7)
Supplement: Supplementary file 1 — Supplementary material 1 (DOC 39 kb) [file 10620_2012_2191_MOESM1_ESM.doc]

**Addendum table 3 Index of suspicion of malignancy^6^**

|  |  | | ***Non specific*** | ***Suggestive*** | ***Strong suspicion*** |
| --- | --- | --- | --- | --- | --- |
| **Clinical symptoms** | | | Weight loss<5kg | Jaundice | >10kg weight loss |
|  |  | | Mild to moderate pain alone |  | +/- severe pain |
| **Ca 19.9** | | | <34 kU/l = normal | >34 and <300 kU/l | ≥ 300 kU/l |
| **Preoperative cytology or histology** | | | Benign | Atypical | Malignant |
|  |  | |  |  |  |
| **Imaging** | | | **Non specific** | **Sugg chronic pancreatitis** | **Suggestive neoplasm** |
|  | **US, CT, MRI** | | Mass (solid / cystic) | Calcifications, pseudocyst | Double duct dilation |
|  |  | | Focal enlargement | Parenchymal atrophy | Blunt stenosis CBD or PD stenosis |
|  |  | | Single duct dilation | Tapering stenosis PD or CBD | Abnormal portal venous Doppler / encasement |
|  |  | | Thickening CBD wall | Diffuse enlargement / rim | Regional adenopathy |
|  |  | |  | PD irregularities | Infiltration |
|  | **ERCP** | | Single duct dilation | CBD stenosis long, tapered, smooth | CBD stenosis (short, irregular, shouldering, excentric, complete) |
|  |  | |  | PD stenosis multiple, short, side branches | PD stenosis (single, >1cm, irregular, no side branche) |
|  |  | |  |  | Double duct sign |
|  | | **Endoscopic US** | Single duct dilatation | Parenchymal / ductal criteria | Mass |
|  |  | | Thickening CBD wall |  | Lymphadenopathy |
|  |  | |  |  | Vascular involvement |

PD: pancreatic duct, CBD: common bile duct
