# Supplementary material for: Prevalence of Autoimmune Pancreatitis and Other Benign Disorders in Pancreatoduodenectomy for Presumed Malignancy of the Pancreatic Head
Source: Dig Dis Sci. 2012 May 16;57(9):2458–65. doi: 10.1007/s10620-012-2191-7 (PMC3428528; doi:10.1007/s10620-012-2191-7)
Supplement: Supplementary file 2 — Supplementary material 2 (DOC 33 kb) [file 10620_2012_2191_MOESM2_ESM.doc]

**Addendum Table 4 HISORT and Asian Diagnostic Criteria (summarized)** [17,18]

| ***Criterion*** |  |  |
| --- | --- | --- |
| **Histology** |  | Lymphoplasmacytic sclerosing pancreatitis and  > 10 IgG4 positive plasmacells / high power field |
| **Imaging** | Typical | Diffusely enlarged pancreas with rim  Diffusely irregular pancreatic duct (PD) |
|  | Other | Focal mass or enlargement, focal PD stricture |
|  |  | Atrophy, calcification, pancreatitis |
| **Serology** | IgG4 > 1.40 g/L | Asian: IgG > 17.0 g/L, presence of autoantibodies (ANA, RF) |
| **Other Organ involvement** |  | Biliary strictures, salivary glands, mediastinal lymphadenopathy, retroperitoneal fibrosis |
| **Response to steroid therapy** |  |  |

*In HISORt criteria system diagnosis is made either if histology alone is positive (resection or pancreatic core biopsy), or if combination typical imaging and elevated IgG4, or if unexplained pancreatic disease with elevated IgG4 or other organ involvement and response to steroids. In Asian criteria typical imaging is mandatory either in combination with serology (IgG4, IgG and RF or ANA) or histology of pancreatic biopsy lesions or histology alone if ipositive in resected pancreas. Response to steroids is conducted only if typical imaging is present and after negative work up for pancreatic cancer. Other organ involvement is not included in Asian criteria.*
